# Systematic evaluation of predictors for binding free energy changes upon mutations in protein complexes

**DOI:** 10.1093/bib/bbaf645

**Published:** 2025-12-08

**Authors:** Yu Zhang, Yunjiong Liu, Yulin Zhang, Ziyang Wang, Xiaoli Lu, Shengxiang Ge, Xiaoping Min

**Affiliations:** Institute of Artificial Intelligence, School of Informatics, Xiamen University, No. 4221, Xiang'an South Road, Xiang'an District, Xiamen City, 361005, Fujian Province, China; National Institute of Diagnostics and Vaccine Development in Infectious Diseases, National Innovation Platform for Industry-Education Integration in Vaccine Research, NMPA Key Laboratory for Research and Evaluation of Infectious Disease Diagnostic Technology, Xiamen University, No. 4221, Xiang'an South Road, Xiang'an District, Xiamen City, 361005, Fujian Province, China; State Key Laboratory of Vaccines for Infectious Diseases, Xiang An Biomedicine Laboratory, School of Public Health, Xiamen University, No. 4221, Xiang'an South Road, Xiang'an District, Xiamen City, 361005, Fujian Province, China; Institute of Artificial Intelligence, School of Informatics, Xiamen University, No. 4221, Xiang'an South Road, Xiang'an District, Xiamen City, 361005, Fujian Province, China; National Institute of Diagnostics and Vaccine Development in Infectious Diseases, National Innovation Platform for Industry-Education Integration in Vaccine Research, NMPA Key Laboratory for Research and Evaluation of Infectious Disease Diagnostic Technology, Xiamen University, No. 4221, Xiang'an South Road, Xiang'an District, Xiamen City, 361005, Fujian Province, China; State Key Laboratory of Vaccines for Infectious Diseases, Xiang An Biomedicine Laboratory, School of Public Health, Xiamen University, No. 4221, Xiang'an South Road, Xiang'an District, Xiamen City, 361005, Fujian Province, China; National Institute of Diagnostics and Vaccine Development in Infectious Diseases, National Innovation Platform for Industry-Education Integration in Vaccine Research, NMPA Key Laboratory for Research and Evaluation of Infectious Disease Diagnostic Technology, Xiamen University, No. 4221, Xiang'an South Road, Xiang'an District, Xiamen City, 361005, Fujian Province, China; State Key Laboratory of Vaccines for Infectious Diseases, Xiang An Biomedicine Laboratory, School of Public Health, Xiamen University, No. 4221, Xiang'an South Road, Xiang'an District, Xiamen City, 361005, Fujian Province, China; Institute of Artificial Intelligence, School of Informatics, Xiamen University, No. 4221, Xiang'an South Road, Xiang'an District, Xiamen City, 361005, Fujian Province, China; National Institute of Diagnostics and Vaccine Development in Infectious Diseases, National Innovation Platform for Industry-Education Integration in Vaccine Research, NMPA Key Laboratory for Research and Evaluation of Infectious Disease Diagnostic Technology, Xiamen University, No. 4221, Xiang'an South Road, Xiang'an District, Xiamen City, 361005, Fujian Province, China; State Key Laboratory of Vaccines for Infectious Diseases, Xiang An Biomedicine Laboratory, School of Public Health, Xiamen University, No. 4221, Xiang'an South Road, Xiang'an District, Xiamen City, 361005, Fujian Province, China; Information and Networking Center, Xiamen University, No. 4221, Xiang'an South Road, Xiang'an District, Xiamen City, 361005, Fujian Province, China; National Institute of Diagnostics and Vaccine Development in Infectious Diseases, National Innovation Platform for Industry-Education Integration in Vaccine Research, NMPA Key Laboratory for Research and Evaluation of Infectious Disease Diagnostic Technology, Xiamen University, No. 4221, Xiang'an South Road, Xiang'an District, Xiamen City, 361005, Fujian Province, China; State Key Laboratory of Vaccines for Infectious Diseases, Xiang An Biomedicine Laboratory, School of Public Health, Xiamen University, No. 4221, Xiang'an South Road, Xiang'an District, Xiamen City, 361005, Fujian Province, China; Institute of Artificial Intelligence, School of Informatics, Xiamen University, No. 4221, Xiang'an South Road, Xiang'an District, Xiamen City, 361005, Fujian Province, China; National Institute of Diagnostics and Vaccine Development in Infectious Diseases, National Innovation Platform for Industry-Education Integration in Vaccine Research, NMPA Key Laboratory for Research and Evaluation of Infectious Disease Diagnostic Technology, Xiamen University, No. 4221, Xiang'an South Road, Xiang'an District, Xiamen City, 361005, Fujian Province, China; State Key Laboratory of Vaccines for Infectious Diseases, Xiang An Biomedicine Laboratory, School of Public Health, Xiamen University, No. 4221, Xiang'an South Road, Xiang'an District, Xiamen City, 361005, Fujian Province, China

**Keywords:** protein complex mutations, binding free energy change (Δ Δ *G*), predictor evaluation, independent evaluation set

## Abstract

The prediction of binding free energy changes ($\Delta \Delta G$) caused by mutations in protein complexes is crucial for understanding disease mechanisms and designing antibodies. Approximately 60% of pathogenic missense mutations lead to functional abnormalities by disrupting molecular interactions. However, although existing $\Delta \Delta G$ predictors exhibit strong performance in benchmarks, they suffer from inadequate generalization, a misalignment between evaluation metrics and practical needs, and poor adaptability to complex mutation scenarios. This study systematically assessed eight mainstream predictors, covering both physical energy function-based and machine learning-based methods, and constructed an independent evaluation set. This study employed multi-dimensional metrics, including regression accuracy and classification capability, while also analyzing the performance variations of predictors across different mutation types, stability categories, and microenvironments of protein mutation sites. The results indicate that >60% of predictors (5 out of 8) predictors exhibit a systematic bias toward overestimating mutational instability. In the three-class classification task, predictors demonstrate a limited ability to identify stabilizing mutations ($\Delta \Delta G< -0.5$ kcal/mol), with recall rates <0.1 for this class, and overall predictive efficacy depends on the protein local structure. In summary, this study reveals the limitations of current $\Delta \Delta G$ predictors in terms of generalization and adaptability to complex scenarios, thus providing a reference for the optimization and practical application of $\Delta \Delta G$ prediction methods. It suggests that future breakthroughs can be achieved by constructing balanced and standardized datasets alongside developing local–global fusion algorithms.

## Introduction

Amino acid mutations affect structure and physicochemical properties of protein complexes by altering the local microenvironments or global conformations [1, 2]. It has been shown that $\sim $60% of pathogenic missense mutations disrupt critical molecular interactions [3], such as salt bridge breakage or hydrogen bond reorganization [4], thereby reducing protein binding affinity [5, 6], ultimately leading to functional abnormalities. Protein misfolding caused by mutations is a key pathogenic mechanism in many diseases [7]. A crucial indicator for detecting pathogenic mutations is the change in binding free energy ($\Delta \Delta G$) before and after the mutations [8–11]. Accurate quantification of $\Delta \Delta G$ not only provides key evidence for understanding the mechanisms of genetic diseases like neurodegenerative disorders but also lays the foundation for designing antibodies for diseases such as cancer [12]. Therefore, the development of high-precision predictors for protein complex $\Delta \Delta G$ is critical for advancing precision medicine and enabling personalized treatment.

Currently, in terms of implementation, $\Delta \Delta G$ prediction methods can be classified into two main types: sequence-based and structure-based prediction methods. Sequence-based methods, which mainly utilize sequence information and evolutionary features for prediction, have a broader range of application scenarios but relatively lower accuracy [13–15]. In contrast, structure-based methods predict $\Delta \Delta G$ by modeling atomic interactions within the 3D conformation of proteins, achieving relatively higher prediction accuracy. However, it is important to note that the accuracy of these methods is highly dependent on the quality of the input structures. Over the past three decades, many predictors have been developed [16, 17], with structure-based methods dominating. During this period, the performance of predictor reported in recent literature has shown an upward trend [18, 19], due to the expansion and improvement of dataset scale and quality, as well as the iteration of algorithms. Among them, machine learning methods have improved prediction performance, owing to their powerful feature learning capabilities [16]. This improvement benefits from continuous innovation in feature engineering techniques, where feature sources have expanded from basic physicochemical features to integrating multi-dimensional information features, such as coevolutionary signals [20] and conformational dynamics [21]. Furthermore, the performance of machine learning models relies on high-quality structured datasets [22]. SKEMPI v2.0 [23], as a comprehensive and widely used benchmark dataset in this field, provides indispensable value for model training and evaluation.

However, it is worth exploring whether these predictors, which perform excellently in benchmark tests, can maintain reliable generalizability and accuracy in real-world application scenarios, such as facing novel protein families, complex multi-point mutations, and challenges in user interaction or deployment environments [24–27]. Existing research reveals three key challenges in the current field [16, 28–32]. First, the data overlap issue in datasets leads to an overestimation of predictor generalization ability. For example, datasets like SKEMPI v2.0, AB-Bind, etc. [23, 33, 34] contain similar homologous proteins or mutation samples. This means that excellent performance on common test subsets may not truly reflect a predictor’s ability to handle novel protein mutations. Second, there is a misalignment between predictor evaluation metrics and practical needs. Current prediction studies primarily focus on regression task metrics (such as RMSE, PCC, etc.). However, biological researchers in practical applications may place more emphasis on pragmatic judgments, including whether a mutation significantly weakens or completely disrupts binding; the robustness of prediction results to input perturbations; and the stability of predictors in real workflows. Finally, different predictors exhibit variations in computational resource requirements, data preprocessing pipelines, software runtime stability, and result definition [18, 35, 36]. The above differences undermine the credibility of prediction results through two pathways: operational risks (e.g. human data preprocessing introducing bias) and environmental dependencies [37] (e.g. hardware limitations preventing execution).

While previous benchmarking studies have quantified systematic biases in $\Delta \Delta G$ predictions [38], the performance and limitations of state-of-the-art predictors in complex practical scenarios such as multi-point mutations, local structural microenvironments, and antibody development remain underexplored. To evaluate the performance of $\Delta \Delta G$ prediction methods, this study constructed an evaluation set containing various mutation types. It includes single-point mutations, multi-point mutations, mutations enhancing or weakening binding stability, as well as samples from different categories like general protein–protein complexes and antigen–antibody complexes. For evaluation predictor selection, this study primarily focused on structure-based prediction methods, selecting the widely used classical energy calculation tool FoldX 5.0 [39], along with five open-source and high-performing structure-based predictors: DDMut-PPI [18], DDAffinity [19], GearBind [35], Prompt-DDG [40], and Light-DDG [41]. Additionally, to evaluate the performance gains from structural information, we included two sequence-based predictors AttABseq [36] and SAAMBE-SEQ [13] as comparative baseline. This selection strategy not only evaluates the latest performance of structure-based prediction methods but also clarifies the value of structural information through comparison with sequence-based methods. All predictor selections were based on their methodological representativeness, algorithmic novelty, and practical application value. The core objectives of this study focus on the generalization performance, classification capability, and application effectiveness of the predictors in antibody optimization scenarios. Furthermore, given evidence suggesting that prediction accuracy may be influenced by local structural microenvironments [42–44], we evaluated the prediction performance of each predictor across different structural categories. Subsequently, based on $\Delta \Delta G$ thresholds, the dataset was divided into three classes (enhancing/neutral/weakening) to evaluate the qualitative classification accuracy of the predictors regarding mutation binding effects. Finally, addressing the significant challenges of escape mutation prediction and affinity optimization in antibody development [45–47], the performance of the predictors in single-/multi-point mutations of antigen–antibody complexes was evaluated. Through systematic evaluation, this study reveals the performance limitations of current predictors and provides a reference for solving the aforementioned challenges.

## Materials and Methods

### Construction of the evaluation set

To ensure independence of the evaluation, this study constructed a novel evaluation set. The data primarily came from three public databases: ATLAS [48], ThermoMutDB [49], and PROXiMATE [50]. In addition, experimental data such as antigen–antibody interactions were supplemented from relevant literature [51–54]. All samples were rigorously screened to ensure no overlap with the predictors’ training sets (SKEMPI v2.0 and AB-Bind).

A total of 1753 experimental samples were selected, all of which include protein–protein complex structures, mutation annotations, and $\Delta \Delta G$ values. The corresponding complex structure files were obtained via the SYNC protocol from RCSB Protein Data Bank (PDB) [55]. Details of the evaluation set are shown in Table 1. First, two sets, PPI and ABAG, were constructed from the 1753 experimental samples. ABAG is a subset of PPI, representing the antigen–antibody complex mutation dataset, while PPI denotes the general protein–protein complex mutation dataset. Second, the PPI dataset covers 118 protein–protein complex structures, and the ABAG dataset covers 26 antigen–antibody complex structures. This division retains the research value of general protein–protein interactions and provides data support for the critical scenario of immune recognition. It also establishes a data foundation for evaluating the performance differences of the predictors across different biological interaction scenarios.

**Table 1:** Detailed information of the evaluation set

Feature category	PPI ($n=1753$)	ABAG ($n=921$)
Mutation type distribution		
Single-point mutations	1029 (58.7%)	277 (30.1%)
Multi-point mutations	724 (41.3%)	644 (69.9%)
• 2–5 point mutations ($2 \leq m \leq 5$)	410 (23.4%)	336 (36.5%)
• 6+ point mutations ($m \geq 6$)	314 (17.9%)	308 (33.4%)
$\Delta \Delta G$ Functional Impact		
Strong mutations ($|\Delta \Delta G| $ > 0.5)	1008 (57.5%)	547 (59.4%)
• $\Delta \Delta G $ > 0.5 (Destabilizing)	755 (43.1%)	458 (49.7%)
• $\Delta \Delta G $ < −0.5 (Stabilizing)	253 (14.4%)	89 (9.7%)
Weak mutations ($|\Delta \Delta G|$ $ \leq 0.5$)	745 (42.5%)	374 (40.6%)

### Introduction to evaluated $\Delta \Delta G$ predictors

This study systematically evaluated eight $\Delta \Delta G$ predictors in protein complexes, including traditional physical energy function methods and machine learning methods. The input parameter characteristics and functions of these predictors are detailed in Supplementary, Section S1. Among the eight evaluation predictors, DDMut-PPI defines $\Delta \Delta G$ as $\Delta \Delta G= -$($\Delta $Gm − $\Delta $Gw), which is opposite to the $\Delta \Delta G$ definition used by other predictors ($\Delta \Delta G$ = $\Delta $Gm − $\Delta $Gw). Therefore, we inverted the sign of the $\Delta \Delta G$ values predicted by DDMut-PPI to align them with the predictions from other predictors. We selected the model parameters that yielded the best performance as reported in the original publications of all predictors in the evaluation. The introduction of the evaluation predictors is as follows:

- **DDMut-PPI** [18] is a deep learning model based on graph convolutional networks (GCNs). Its core innovation lies in constructing a PPI interface graph: nodes represent interface residues, edges represent the 10 types of molecular interactions defined by Arpeggio, and structural features such as FoldX energy terms and residue depth are combined to form a multidimensional feature space.
- **DDAffinity** [19] is a deep learning model based on a spatial-sequence message passing neural network. A k-nearest neighbor residue graph is constructed to integrate rationalized properties and edge features, and a three-channel encoder is then employed to capture local conformation, linear interactions, and residue synergistic effects, respectively.
- **GearBind** [35] models protein complex interactions by constructing a multi-relational interface atom graph. It employs a three-level geometric information transmission mechanism: atom-level feature updating is performed via relational GCNs; edge-level angle information is transmitted via line graphs; and residue-level global interactions are captured via geometric attention mechanisms.
- **AttABseq** [36] is a deep learning model that uses an attention mechanism to predict changes in antigen–antibody binding affinity caused by point mutations, based solely on sequence information. It adopts an end-to-end architecture with three core modules: an embedding block, an attention block, and a prediction block.
- **SAAMBE-SEQ** [13] is a sequence-based machine learning method for predicting how single mutations affect protein–protein binding affinity. Developed using the Gradient Boosting Decision Tree algorithm and trained on 2398 mutations from the SKEMPI v2.0 database.
- **FoldX 5.0** [39] is based on the classical physical energy function method.
- **Prompt-DDG** [40] is a hierarchical prompt-learning framework. It employs a three-layer prompt codebook to discretize and record common microenvironment patterns across different structural scales. By encoding microenvironment differences into structure-aware signals, the framework significantly improves $\Delta \Delta G$ prediction performance.
- **Light-DDG** [41] is a lightweight structure-aware Transformer framework designed to efficiently predict $\Delta \Delta G$ through knowledge distillation and large-scale dataset augmentation. The framework integrates an iterative Shapley value mutation interpreter for analyzing synergistic effects and employs a preference-guided mutation search to achieve unsupervised antibody optimization, thus offering an efficient solution for protein design.

### Evaluation metrics

This study established a multidimensional evaluation framework to assess the predictive performance of the predictors, which covers three main aspects: regression accuracy, classification discriminative power, and performance across different mutation types. The framework employs an energy threshold of $\pm $0.5 kcal/mol as the stability classification criterion [56].

For regression accuracy assessment, we utilized the Pearson correlation coefficient (r), mean absolute error (MAE), and root mean squared error (RMSE) as evaluation metrics. For classification performance evaluation, both binary and ternary classifications were conducted based on the aforementioned threshold: binary classification employed recall, specificity, precision, F1-score, and AUC metrics to evaluate the discriminative ability between significant effect mutations/neutral mutations; ternary classification utilized granular metrics including per-class accuracy (Accuracy$_{c}$), per-class recall (Recall$_{c}$), per-class precision (Precision$_{c}$), and per-class F1-score (F1$_{c}$) to assess the recognition performance of the three mutation categories, respectively, with comprehensive performance quantification through Kappa coefficient, macro-averaged F1 (Macro-F1), and weighted F1 (Weighted$_{F}1$).

To validate predictor adaptability across diverse scenarios, data were further stratified by mutation type (single-point/multi-point), interaction type (antigen–antibody/other complexes), and the aforementioned stability classification, enabling systematic analysis of performance variations across scenarios. Detailed computational formulas for all metrics are provided in Supplementary, Section 2.

## Results

### Overall performance of predictors

To reveal the performance of eight $\Delta \Delta G$ predictors, this study evaluated 1753 PPI mutants and 921 ABAG mutants. The relevant results are shown in Table 2. The top one predictor, based on their performance in the PPI and ABAG sets, is DDMut-PPI. Although DDMut-PPI achieves the highest $r$-value in both datasets, its prediction accuracy ($r <$ 0.5) still has significant room for improvement. Additionally, DDMut-PPI outperforms the traditional method FoldX 5.0, with gains in $r$-value of 0.4192 and 0.2912 on the PPI and ABAG datasets, respectively.

**Table 2:** Regression metrics for each predictor on the PPI and ABAG datasets. Balanced sampling was employed: five independent random samplings (35 mutations each) were performed on the three PDB structures (6M0J, 7C01, and 7KMG), with the final metric being the average of the five iterations

Metric	DataSet	Predictor					
		DDMut-PPI	Light	Prompt	GearBind	FoldX	DDAffinity
Pearson $r$	PPI	**0.4408**	0.4372	0.3162	0.3105	0.0216	−0.0440
	ABAG	**0.4701**	0.4254	0.3822	0.2160	0.1789	−0.1467
MAE	PPI	1.1845	**1.0721**	1.1499	1.2545	2.0249	1.3020
	ABAG	1.2009	**1.0559**	1.2641	2.0795	1.3593	1.4436
RMSE	PPI	1.8367	**1.6107**	1.7712	1.7928	3.5073	1.9590
	ABAG	1.7854	**1.6033**	1.8888	3.4517	2.0269	2.1211

Bold text indicates the best performance for each metric-dataset combination, while underlined text indicates the second-best performance.

**Figure 1:**
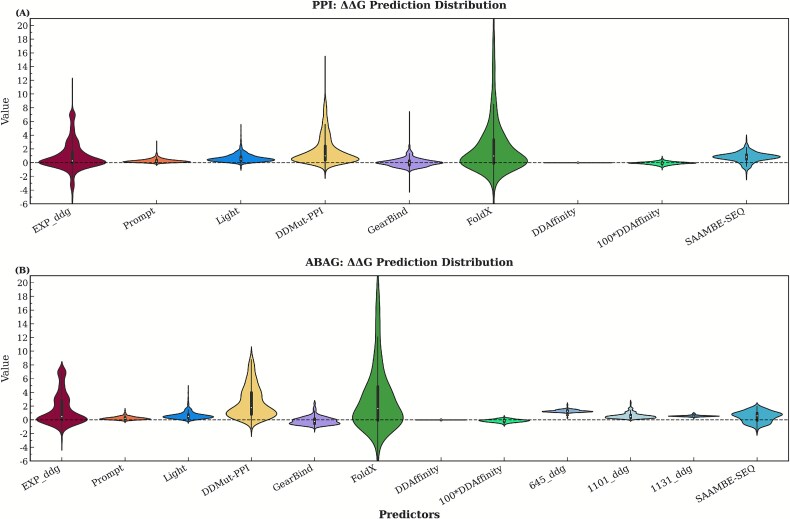
(A) Predicted versus true values for the PPI dataset; (B) predicted versus true values for the ABAG dataset. Note: 100*DDAffinity corresponds to 100 times the DDAffinity predicted value (just for visualization); 645${\_}$ddg, 1101${\_}$ddg, and 1131${\_}$ddg are the three subpredictors included in the AttABseq predictor; The SAAMBE-SEQ predictor contains only the predicted values for single-point mutation data.

Key systematic biases are revealed through analysis of the $\Delta \Delta G$ distribution, as illustrated in Fig. 1. DDMut-PPI, Prompt-DDG, and Light-DDG all exhibit a systematic positive bias in their predictions, meaning they overestimate the $\Delta \Delta G$ values. This overestimation is particularly pronounced in antigen–antibody complexes. These deviations may be attributed to the prevalence (61%) of unstable mutations within the SKEMPI v2.0 training set. Notably, the sequence-based predictor AttABseq is excluded from further analysis because it holistically overestimated $\Delta \Delta G$ values, as illustrated in Fig. 1B.

### Performance of predictors under varying mutation complexity

To systematically evaluate the impact of mutational complexity on prediction performance, we conducted a stratified analysis of the ABAG and PPI datasets based on the number of mutation points. The limitations of synergy modeling generally result in poor performance of predictors under multi-point mutation. As shown in our stratified analysis (Table 3), the regression performance of predictors degrades with increasing mutational complexity. The sequence-based SAAMBE-SEQ underperforms compared with structure-based models, highlighting the critical importance of structural information.

**Table 3:** Comparison of regression performance of predictors under different mutation complexity scenarios.

Predictor	MUTANT	PPI			ABAG		
		Pearson $r$	MAE	RMSE	Pearson $r$	MAE	RMSE
Prompt	Single-point	0.4034	0.9236	1.3774	0.5045	1.0538	1.5178
	Multi-point	0.2008	2.0015	2.9409	0.2577	1.9391	2.8892
	Multi-2–5	0.1867	1.5712	2.4432	0.1993	1.4096	2.2612
	Multi$\geq $6	0.2689	2.5635	3.4854	–	—	–
Light	Single-point	0.5006	0.9166	1.3092	0.6484	0.8313	1.1338
	Multi-point	0.2249	1.937	2.7959	0.1824	1.8747	2.7601
	Multi-2–5	0.2459	1.5665	2.3075	0.1584	1.4047	2.1417
	Multi$\geq $6	0.5048	2.4208	3.3274	–	–	–
DDMut-PPI	Single-point	0.3543	0.9862	1.4659	0.6351	0.808	1.1272
	Multi-point	0.3842	2.5851	3.2441	0.342	2.532	3.1523
	Multi-2–5	0.4809	1.6666	2.2654	0.5136	1.4294	1.8402
	Multi$\geq $6	0.1780	3.7844	4.1910	–	–	–
GearBind	Single-point	0.3073	0.9904	1.4168	0.4113	1.0861	1.5150
	Multi-point	0.0644	2.2809	3.2431	−0.1189	2.2799	3.2994
	Multi-2–5	0.206	1.802	2.5974	−0.0701	1.7247	2.5971
	Multi$\geq $6	−0.0817	2.9064	3.9295	–	–	–
Foldx	Single-point	0.0224	1.5771	2.6924	0.2342	1.3808	1.9669
	Multi-point	0.076	4.5056	6.3215	0.1267	4.4234	6.2044
	Multi-2–5	0.0663	3.0084	4.1975	0.2339	2.6585	3.4949
	Multi$\geq $6	−0.0595	6.4606	8.3147	–	–	–
DDAffinity	Single-point	−0.0273	1.0155	1.5197	−0.0782	1.2418	1.7761
	Multi-point	−0.0845	2.0699	3.1036	−0.0541	2.0016	3.0573
	Multi-2–5	−0.0502	1.6251	2.5797	−0.0874	1.4444	2.3887
	Multi$\geq $6	−0.0861	2.6506	3.6771	–	–	–
SAAMBE-SEQ	Single-point	−0.1593	1.3021	1.7899	−0.1659	1.3595	1.8417

Note: This table compares regression performance of $\Delta \Delta G$ predictors across different mutation complexity scenarios on PPI and ABAG datasets. Categories 2–5 and $\geq $6 represent multiple-point mutation samples containing 2 to 5, and 6 or more mutation points, respectively. The results regarding multiple-point mutations depend on specific structures (7C01 and 7KMG).

Specifically, performance becomes poorer as the number of mutation points increases. This trend is reflected in the performance of DDMut-PPI, which achieves relatively high $r$-value for complexes with 2–5 mutations but exhibits a significant performance drop when the number of mutations reaches $\geq $ 6. Conversely, Light-DDG displays the opposite behavior: it performs well in the single-point mutation scenario and its performance declines for complexes with 2–5 mutations, which aligns with the expected trend. However, its $r$-value increases for complexes with $\geq $ 6 mutations, deviating from the general observation that predictive performance deteriorates with increasing mutational complexity. This divergence can be attributed to the inherent adaptability of each algorithm’s design to different levels of mutational complexity. DDMut-PPI is particularly suited for scenarios involving local synergistic effects. Its architecture employs a GCN to process interaction interfaces, integrates ProtT5 embeddings and Arpeggio interaction features, and predicts multi-point mutations by summing single-point mutation effects. This design excels at capturing local conformational changes and synergistic effects in 2–5 point mutations. However, when mutations reach $\geq $ 6 points, global correlations dominate, and the summation strategy fails to capture these complex interactions, leading to performance degradation. In contrast, Light-DDG, built on a Transformer framework, captures global evolutionary information through ProtT5 embeddings and iteratively estimates the marginal contribution of mutations. This design reduces its susceptibility to being trapped in local optima and enhances its adaptability to complex global synergistic scenarios. Specifically, in single-point mutation, global information is sufficient to support prediction. When the number of mutations is $\geq $ 6, complex synergistic effects rely more on global associations, highlighting its architectural advantages and consequently improving performance. However, with 2–5 point mutations, the impact of local conformational changes on prediction becomes more pronounced, and the architecture’s lack of local structure perception capabilities leads to a decline in performance.

Overall, the aforementioned results reveal a common phenomenon and challenge in current algorithms for predicting complex mutations: within the regression task, the predictive accuracy for multi-point mutation diminishes as the number of mutations increases, although certain predictor exhibit a turning point improvement.

### Performance of predictors in different classification tasks

**Figure 2:**
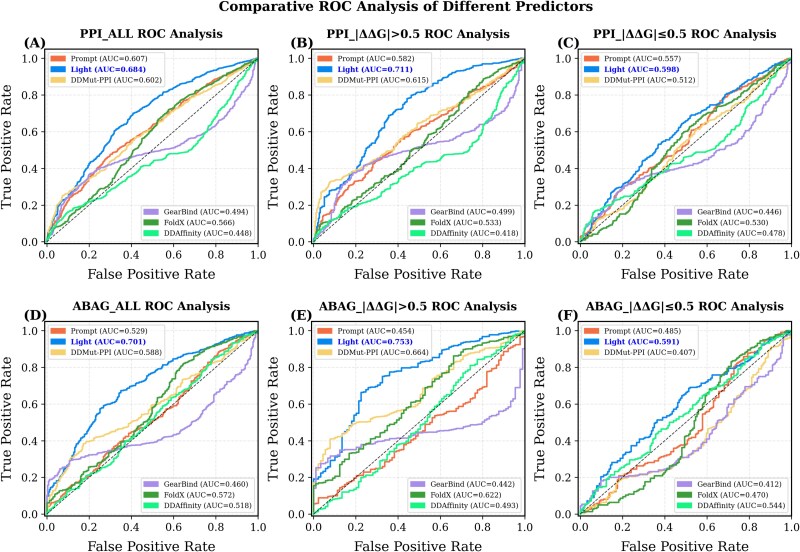
ROC curves for each predictor under different stability classification scenarios. Subfigures (A)–(C) present results on the PPI dataset, while (D)–(F) show corresponding analyses on the ABAG dataset.

To assess the practical utility of predictors in guiding biological decision-making, the performance of protein complex mutation $\Delta \Delta G$ predictors was analyzed in this study through a two-level classification evaluation system. For the binary classification task, $\Delta \Delta G$ was divided into significant effect mutations ($|\Delta \Delta G|$ > 0.5) and neutral mutations ($|\Delta \Delta G|$  $\leq $ 0.5) based on the 0.5 kcal/mol energy threshold recommended in the literature [56]. The results are shown in Fig. 2 and Supplementary, Section S3. Light-DDG maintains the leading AUC value across all six tasks, achieving a highest discrimination of 0.753 in the binary classification task for significant mutations. However, its performance in the binary classification task for neutral mutations is poor. Notably, Prompt-DDG, Light-DDG, and DDMut-PPI exhibit high recall rates across all stability binary classification tasks; however, this advantage is offset by low specificity. Conversely, DDAffinity demonstrates optimal performance in terms of specificity, particularly reaching its highest level within the $|\Delta \Delta G|$  $\leq $ 0.5 subgroup of the PPI and ABAG datasets, but its recall rate is limited.

**Figure 3:**
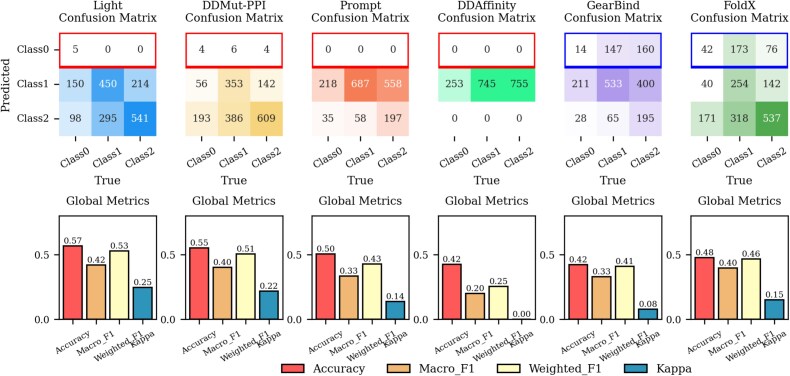
Three-class classification results of the predictor on the PPI dataset.

To analyze more precisely the ability of each predictor to identify different $\Delta \Delta G$ intervals, we further expanded the classification dimension by conducting a three-class classification task. Based on the threshold of 0.5 kcal/mol, both the true and predicted values of $\Delta \Delta G$ for each predictor were subdivided into three classes: Class${\_}$0 ($\Delta \Delta G$ < 0.5), Class${\_}$1 (-0.5 $\leq $  $\Delta \Delta G$  $\leq $ 0.5), and Class${\_}$2 ($\Delta \Delta G$ > 0.5). Subsequently, a three-class classification performance evaluation was conducted for each predictor. The results, illustrating the three-class classification performance of each predictor on the PPI dataset, are presented in Fig. 3. The results for the ABAG dataset are available in Supplementary, Section S4, show consistent trends with the PPI dataset.

Light-DDG outperforms other predictors in all global metrics on both the PPI and the ABAG datasets, with DDMut-PPI ranking second. However, the performance of all predictors remains at a medium-to-low level, not yet reaching the desired state. Examining the confusion matrices of each predictor for classification prediction, it is observed that, except for GearBind and FoldX, the prediction results of other predictors are almost exclusively concentrated in Class${\_}$1 and Class${\_}$2. Specifically, the proportion of samples with predicted values below $-0.5$ is <1%, lower than the 5%–10% proportion of such samples in the actual data. This phenomenon may be related to the distribution characteristics of the training set of the predictors. Statistics on the SKEMPI v2.0 and AB-Bind datasets indicate that the proportion of samples with $\Delta \Delta G$ values < −0.5 in these two training sets is only 5%–10% This may result in the model’s insufficient learning of values within this range. Although GearBind and FoldX predict a significantly higher proportion of Class${\_}$0 samples than other predictors, their prediction accuracy is very low. FoldX, being a physical energy-based model, does not rely on training set training and is therefore unaffected by the distribution of the training set, allowing it to predict more values in this range. GearBind as a machine learning model requiring supervised training based on the training set, still exhibits a high proportion of predictions with $\Delta \Delta G$ below $-0.5$, which is speculated to be related to its additional pretraining on the CATH dataset involving natural/mutated structure comparison. Considering the performance of metrics across different categories, all predictors show poor accuracy, recall, and F1 scores in Class${\_}$0 ($\Delta \Delta G<$0.5). Conversely, these metrics for each predictor improve to some extent in Class${\_}$1 (-0.5 $\leq $  $\Delta \Delta G$  $\leq $ 0.5) and Class${\_}$2 ($\Delta \Delta G>$0.5).

### Performance of predictor on local structural microenvironments

**Figure 4:**
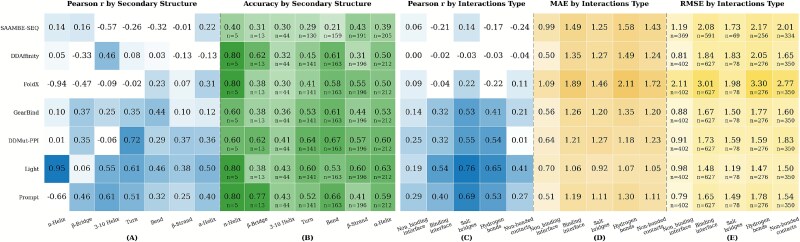
Characterization of the local structural environments for mutation sites. Subfigures (A) and (B) present the statistical results based on the secondary structure classification; subfigures (C)–(E) display the statistical data for mutations at binding interfaces versus nonbinding interfaces, as well as the results for the types of interactions.

We conducted a detailed investigation of the local structural context surrounding mutation sites. This study classifies and statistically analyzes the secondary structures of wild-type amino acids at mutation sites based on DSSP (Dictionary of Secondary Structure of Proteins) [44]. As a standard tool in protein structure biology, DSSP classifies residues into eight types of topological conformations based on hydrogen bond network patterns: H ($\alpha $-Helix), B ($\beta $-Bridge), E ($\beta $-Strand), G (3-10 Helix), I ($\pi $-Helix), T (Turn), S (Bend), and C (Coil/Loop). Additionally, we subdivided the samples based on whether the mutation site is located at a binding interface into interface and noninterface mutations [38]. Similar to the classification of physicochemical properties of protein binding pockets in the CASF series of studies [57–59], for mutations occurring at binding interfaces, we further categorized them into three types of interactions: salt bridges, hydrogen bonds, and nonbonded contacts. The relevant results are shown in Fig. 4.

As shown in Fig 4A and B, no predictor demonstrated consistent superiority across these secondary structure types. The correlation coefficients and ternary classification accuracy of each predictor varied considerably across different structures, indicating that their applicability is limited to specific structural contexts. Among these predictors, DDMut-PPI exhibited outstanding performance in turn structures, achieving the highest correlation coefficient ($r = 0.72$) among all structure–method combinations. It also showed high classification accuracy and the lowest MAE in turn conformations, suggesting a strong capability in capturing local turn structural features. Furthermore, the results indicated that among the seven secondary structure types, Turns and 3–10 HeliX were relatively easier to predict compared with others, though predictive performance in these categories still requires improvement. To explore potential training data bias concerns, we further analyzed the secondary structure distribution in training datasets (SKEMPI v2.0 and AB-Bind). As shown in Supplementary, Section S5, the training set exhibits a balanced representation across major secondary structure types: $\beta $-Strand (36.0%), $\alpha $-Helix (15.5%), Bend (21.8%), and Turn (19.6%). The evaluation set distribution closely mirrors this pattern, ensuring fair evaluation without structural sampling bias. Notably, the underrepresentation of $\beta $-Bridge (3.7%) and $\pi $-Helix (0.1%) in both datasets explains the poor performance across all predictors for these structural categories, highlighting the need for more diverse structural sampling in future dataset construction. Although $\beta $-Strand has the largest proportion in the training set, the performance of various predictors on it is not optimal, which may be related to the intrinsic physicochemical properties of the structure. Turn, as conformationally constrained short-range structures, has localized hydrogen bonding and steric environments that make computational modeling of mutational effects more direct and stable. In contrast, the stability of $\beta $-Strand relies on long-range hydrogen bonding networks and side-chain packing. The effect of a single mutation can easily propagate through the sheet via cooperative interactions, and this complex long-range mechanism significantly increases the difficulty of accurately predicting its $\Delta \Delta G$ changes.

As shown in Fig 4C–E, noninterface mutations serve as an important control group. Figure 4C shows that the correlation coefficients of all predictors for these mutations were significantly lower than those for other categories. This may be because noninterface mutations do not directly participate in interactions, and the resulting $\Delta \Delta G$ changes are generally small and involve complex mechanisms (e.g. through long-range allosteric effects). In contrast, interface mutations directly affect binding affinity, leading to more pronounced effects that are easier to predict. This confirms that predicting indirect effects remains a major challenge for current methods. The performance of all predictors is highly dependent on the structural environment of the mutation site. There is no single “universal” predictor that performs optimally in all scenarios. Among the three interaction types-salt bridges, hydrogen bonds, and nonbonded contacts-predictors generally performed better for salt bridges and hydrogen bonds compared with nonbonded contacts. Notably, Light showed particularly strong performance in scenarios involving specific interactions (hydrogen bonds, salt bridges), whereas all predictors exhibited a general decline in performance in regions dominated by nonbonded contacts. SAAMBE-SEQ, as a sequence-based method, performed poorly-and even showed negative correlation-in most scenarios requiring structural information (e.g. binding interfaces), highlighting the necessity of structural information for accurate $\Delta \Delta G$ prediction.

### Performance evaluation on industrially relevant therapeutic targets

**Figure 5:**
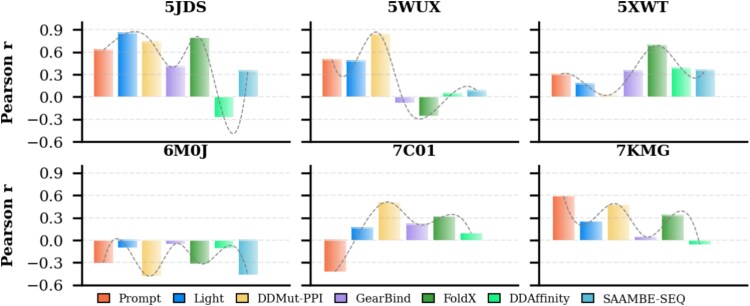
Regression performance of predictors on six protein complexes.

To evaluate the practical utility of predictors in computer-aided drug discovery, we selected six industrially relevant protein complexes as a benchmark test set (detailed information is provided in Supplementary, Section 6). As shown in Fig. 5, the regression performance on these targets reveals context-dependent predictor efficacy. On the 5JDS complex, most predictors performed well, which may be attributed to all mutations being significantly destabilizing. However, on the 6M0J complex, most predictors exhibited opposite trends, likely because only $\sim $4% of mutations in the 6M0J dataset were significantly destabilizing (detailed mutation data for 6M0J are provided in Supplementary, Section S7), consistent with our earlier analytical conclusions. This case-based evaluation confirms that although predictors can demonstrate strong performance on specific therapeutic targets such as PD-L1 and TNF-$\alpha $, their efficacy is highly variable. Predictive performance on complexes with broad biological and industrial significance remains suboptimal. These findings emphasize that future improvements to predictors should include customized benchmarking against high-value, industrially relevant targets to ensure their utility in practical drug discovery applications.

## Discussion and Conclusion

The overall performance of predictors in the current field of protein complex $\Delta \Delta G$ prediction remains limited, with predictors’ insufficient generalization and adaptability to complex scenarios. These common issues primarily stem from three areas. First, a biased training data distribution leads to prediction errors. This is because the training data contains a disproportionately high number of destabilizing mutations, most methods tend to overestimate the disruptive effect of mutations on binding. Second, the ability to capture complex conformations and synergistic effects is insufficient. The performance of predictors declines in multi-point mutation scenarios, making it difficult to model the global coupling mechanisms between mutations. Third, adaptability to specific structural environments is limited. Prediction accuracy is dependent on local secondary structure topology, as evidenced by consistent failures in specific conformations. While some predictors perform well on Turn structures, overall performance is still constrained by the inherent modeling capabilities.

Further analysis of method characteristics indicates that physics-based energy function methods do not rely on training data; however, these methods exhibit limitations in correlation, classification, and error analysis. These limitations reflect their insufficient capacity to describe dynamic conformational changes. Machine learning methods improve the prediction accuracy for single-point mutations through feature learning, but their performance is constrained by the distribution of training data, resulting in weak recognition of stabilizing mutations ($\Delta \Delta G$ < −0.5 kcal/mol) and persistent challenges in modeling synergistic effects for multi-point mutations. In the future, to address the data dependency issue in machine learning, a standardized database covering a wider range of structural types and balancing the ratio of stable to unstable mutations should be established, incorporating high-resolution cryo-EM structures and dynamic conformation data to eliminate the distribution bias between training sets and real-world scenarios. Simultaneously, a unified standard for annotating mutation effects should be established to improve data quality and consistency.

The performance of different predictors on protein secondary structure is dependent on local topological conformations, lacking universal advantages across structures. Higher accuracy is concentrated only in a few specific structures, and significant performance fluctuations are observed between different structures, lacking a unified pattern. This characteristic highlights the limited adaptability of current algorithms to the diversity of protein structures. Regarding future algorithmic development, a multi-scale modeling approach should be adopted: on one hand, leveraging evolutionary and structural prior knowledge from large-scale protein models to enhance generalization capabilities for unknown structures; on the other hand, developing a local–global fusion architecture that balances residue-level interaction details with global conformational dynamics to capture complex synergistic effects. Additionally, integrating multiple types of predictors can complement their respective limitations and enhance prediction robustness. The ultimate goal is to establish a general predictive framework capable of adapting to structural diversity, dynamic conformations, and complex mutation scenarios, providing reliable tools for practical applications such as antibody optimization and disease mechanism analysis. Looking ahead, we anticipate the development of more advanced predictors to address the aforementioned challenges. A particularly promising direction is the integration of physics-based models with machine learning methods, aiming to achieve both speed and accuracy in predicting binding free energy changes upon mutations.

**Key Points**

- We introduced a curated, nonoverlapping evaluation set of 1753 mutations to fairly assess generalizability beyond training data.
- We revealed a common tendency among predictors to overestimate destabilizing effects and perform poorly on stabilizing mutations.
- We showed that predictor accuracy decreases with increasing mutation counts and is highly influenced by local protein secondary structure.
- We identified the need for balanced datasets and novel algorithms that fuse local and global protein information to overcome current limitations.

## Supplementary Material

Supplementary_Materials_bbaf645

## Data Availability

The evaluation set used in this article are available at https://github.com/Sharky-zy/EvaluationSet.
