# Supplementary material for: Systematic evaluation of predictors for binding free energy changes upon mutations in protein complexes
Source: Brief Bioinform. 2025 Dec 8;26(6):bbaf645. doi: 10.1093/bib/bbaf645 (PMC12684732; doi:10.1093/bib/bbaf645)
Supplement: Supplementary_Materials_bbaf645 [file supplementary_materials_bbaf645.pdf]

Supplementary Materials for "Systematic Evaluation of Predictors for Binding Free Energy Changes Upon Mutations in Protein Complexes"

Yu Zhang, Yunjiong Liu, Yulin Zhang, Ziyang Wang, Xiaoli Lu,
Shengxiang Ge, Xiaoping Min

1 Core attribute comparison of the seven predictors

Table 1: Core attribute comparison of the seven predictors. Covers input data types, algorithm advantages, training set foundations, and access methods.

Predictor	Web/Code URL	Input	Training Set
DDMut-PPI	DDMut-PPI Web	Structure of Wild-Type Protein Complex<br>Mutation details	single-point mutation training set: S8338 (based on SKEMPI v2.0)
DDAffinity	DDAffinity GitHub	Structures of Wild-Type and Mutant Protein Complexes<br>Mutation details	SKEMPI 2.0
GearBind	GearBind GitHub	Structures of Wild-Type and Mutant Protein Complexes<br>Mutation details	SKEMPI v2.0
AttABseq	AttABseq GitHub	Sequence of Wild-Type Protein Complex<br>Mutation details	Single-point: AB645 (AB-Bind)<br>S1131 (Skempi 2.0)<br>Multi-point: AB1101 (AB-Bind)
SAAMBE-SEQ	saambe_webserver	Sequence of Wild-Type Protein Complex<br>Mutation details	Single-point: S2398 (Skempi 2.0)
FoldX 5.0	FoldX Suite	Structure of Wild-Type Protein Complex<br>Mutation details	-
Prompt-DDG	Prompt-ΔΔG	Structure of Wild-Type Protein Complex<br>Mutation details	SKEMPI v2.0
Light-DDG	Uni-Anti<br>Light-DDG Web	Structure of Wild-Type Protein Complex<br>Mutation details	SKEMPI v2.0<br>SKEMPI-Aug

2 Formal Definitions of Evaluation Metrics

2.1 Regression Metrics

- **Pearson Correlation Coefficient (r)**:

$$r = \frac{\sum_{i=1}^{n}(x_i - \bar{x})(y_i - \bar{y})}{\sqrt{\sum_{i=1}^{n}(x_i - \bar{x})^2}\sqrt{\sum_{i=1}^{n}(y_i - \bar{y})^2}} \tag{1}$$

Where x_i and y_i are the predicted and true $\Delta\Delta G$ values for the i-th sample, and $\bar{x}$ and $\bar{y}$ are their respective means.

- **Mean Absolute Error (MAE)**:

$$\text{MAE} = \frac{1}{n}\sum_{i=1}^{n}|y_i - x_i| \tag{2}$$

- **Root Mean Squared Error (RMSE)**:

$$\text{RMSE} = \sqrt{\frac{1}{n}\sum_{i=1}^{n}(y_i - x_i)^2} \tag{3}$$

2.2 Metrics for Binary Classification

For the binary classification task (significant effect mutations ($|\Delta\Delta G| > 0.5$) and neutral mutations ($|\Delta\Delta G| \leq 0.5$)):

- **Recall**:

$$\text{Recall} = \frac{\text{TP}}{\text{TP} + \text{FN}} \tag{4}$$

- **Specificity**:

$$\text{Specificity} = \frac{\text{TN}}{\text{TN} + \text{FP}} \tag{5}$$

- **Precision**:

$$\text{Precision} = \frac{\text{TP}}{\text{TP} + \text{FP}} \tag{6}$$

- **F1-score**:

$$\text{F1} = 2 \times \frac{\text{Precision} \times \text{Recall}}{\text{Precision} + \text{Recall}} \tag{7}$$

2.3 Comprehensive Metrics for Ternary Classification

For the ternary classification task (Class 0: $\Delta\Delta G < -0.5$; Class 1: $-0.5 \leq \Delta\Delta G \leq 0.5$; Class 2: $\Delta\Delta G > 0.5$):

- **Macro-Averaged F1-score (Macro-F1)**: The arithmetic mean of the per-class F1-scores:

$$\text{Macro-F1} = \frac{1}{C}\sum_{c=1}^{C}\text{F1}_c \tag{8}$$

  where $C = 3$ is the number of classes.

- **Weighted F1-score (Weighted F1)**: The mean of per-class F1-scores, weighted by class support:

$$\text{Weighted F1} = \frac{\sum_{c=1}^{C} n_c \cdot \text{F1}_c}{\sum_{c=1}^{C} n_c} \tag{9}$$

  where n_c is the number of samples in class c.

- **Cohen's Kappa Coefficient (κ)**: Measures inter-rater agreement, adjusting for chance:

$$\kappa = \frac{p_o - p_e}{1 - p_e} \tag{10}$$

  where p_o is the observed agreement ratio (overall accuracy), and p_e is the probability of chance agreement, calculated from the confusion matrix $\mathbf{M}$:

$$p_o = \frac{\sum_{i=1}^{C} M_{ii}}{N} \tag{11}$$

$$p_e = \frac{\sum_{i=1}^{C}(R_i \cdot C_i)}{N^2} \tag{12}$$

  where N is the total number of samples, R_i is the sum of row i, and C_i is the sum of column i.

3 Performance evaluation of $\Delta\Delta G$ predictors in binary classification using the 0.5 kcal/mol energy threshold

Table 2: Classification performance of predictors compared across different stability scenarios. Classification thresholds: significant effect mutations ($|\Delta\Delta G| > 0.5$ kcal/mol) vs. neutral mutations ($|\Delta\Delta G| \leq 0.5$ kcal/mol).Performance metrics include Recall, F1-score, AUC, MCC, and Specificity.

Dataset	Predictor	MUTANT	Recall	F1	AUC	MCC	Specificity
PPI	Prompt	$\|\Delta\Delta G\| > 0.5$	0.9642	0.8431	0.5823	-0.0004	0.0356
		$\|\Delta\Delta G\| \leq 0.5$	0.9191	0.6642	0.5572	0.0369	0.1022
	Light	$\|\Delta\Delta G\| > 0.5$	0.9841	0.859	0.7107	0.1635	0.083
		$\|\Delta\Delta G\| \leq 0.5$	0.9791	0.68	0.5977	0.0724	0.047
	DDMut-PPI	$\|\Delta\Delta G\| > 0.5$	0.9669	0.8469	0.6155	0.05	0.0553
		$\|\Delta\Delta G\| \leq 0.5$	0.9504	0.6642	0.5116	-0.0338	0.0359
	GearBind	$\|\Delta\Delta G\| > 0.5$	0.5285	0.6205	0.4985	0.0059	0.4783
		$\|\Delta\Delta G\| \leq 0.5$	0.3916	0.4335	0.4459	-0.0483	0.5608
	DDAffinity	$\|\Delta\Delta G\| > 0.5$	0.3311	0.4517	0.4175	-0.0655	0.5968
		$\|\Delta\Delta G\| \leq 0.5$	0.3603	0.424	0.4776	0.0012	0.6409
	Foldx	$\|\Delta\Delta G\| > 0.5$	0.8185	0.7918	0.5332	0.0817	0.2569
		$\|\Delta\Delta G\| \leq 0.5$	0.6449	0.5973	0.53	0.1026	0.4558
ABAG	Prompt	$\|\Delta\Delta G\| > 0.5$	0.9563	0.8911	0.4545	-0.0398	0.0225
		$\|\Delta\Delta G\| \leq 0.5$	0.8909	0.7179	0.4846	0.0688	0.1558
	Light	$\|\Delta\Delta G\| > 0.5$	0.9913	0.9126	0.7527	0.1617	0.0674
		$\|\Delta\Delta G\| \leq 0.5$	0.9864	0.7394	0.5907	0.0448	0.026
	DDMut-PPI	$\|\Delta\Delta G\| > 0.5$	0.9825	0.9045	0.6641	0.0138	0.0225
		$\|\Delta\Delta G\| \leq 0.5$	0.9545	0.7229	0.4068	-0.0698	0.0195
	GearBind	$\|\Delta\Delta G\| > 0.5$	0.4432	0.5759	0.4418	-0.0379	0.5056
		$\|\Delta\Delta G\| \leq 0.5$	0.2273	0.3125	0.4123	-0.1083	0.6753
	DDAffinity	$\|\Delta\Delta G\| > 0.5$	0.2402	0.3697	0.4928	-0.0538	0.6966
		$\|\Delta\Delta G\| \leq 0.5$	0.3045	0.4073	0.5437	0.0345	0.7273
	Foldx	$\|\Delta\Delta G\| > 0.5$	0.8472	0.8575	0.6217	0.1759	0.3371
		$\|\Delta\Delta G\| \leq 0.5$	0.6	0.5986	0.47	0.0221	0.4221

4 Three-class classification performance of $\Delta\Delta G$ predictors on the ABAG dataset

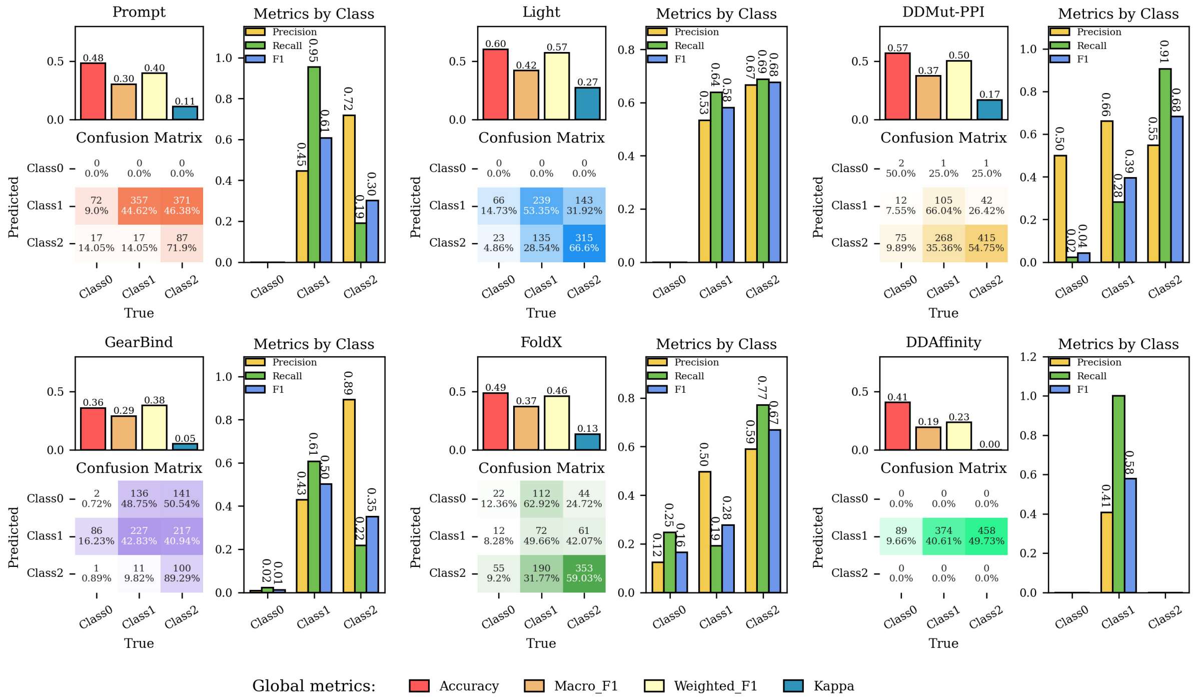

Figure 1: Three-class classification results of the predictor on the ABAG dataset. The rows of the confusion matrix represent the predicted labels (classification results generated by each predictor), while the columns represent the true labels (experimentally measured classification results).

5 Secondary structure distribution in training set and our evaluation sets

Table 3: The table summarizes the proportional occurrence of seven secondary structure elements—H (α-Helix), E (β-Strand), B (β-Bridge), G (3-10 helix), I (π-Helix), T (Turn), and S (Bend)—within the filtered training set (derived from SKEMPI v2.0 and AB-Bind) and the independent test set used for evaluation. Residues classified as coils/loops (C) were excluded from this analysis to focus on defined structural contexts.

Secondary Structure	Training Set (SKEMPI v2.0 + AB-Bind)	Evaluation Set (Ours)
β-Strand (E)	36.0% (703/1953)	25.3% (196/774)
α-Helix (H)	15.5% (303/1953)	27.4% (212/774)
Bend (S)	21.8% (426/1953)	21.1% (163/774)
Turn (T)	19.6% (382/1953)	18.2% (141/774)
3-10 Helix (G)	3.3% (64/1953)	5.7% (44/774)
β-Bridge (B)	3.7% (73/1953)	1.7% (13/774)
π-Helix (I)	0.1% (2/1953)	0.6% (5/774)

6 Detailed information on six industrially relevant protein complexes

Table 4: Overview of six industrially relevant protein complexes.

PDB ID	Complex Description	Therapeutic Area	Sample size
5JDS	PD-L1 / Nanobody	Cancer Immunotherapy	12
5WUX	TNF-α / Certolizumab Fab	Autoimmune Disease	12
5XWT	PTPδ / SALM5	Neuroscience	13
6M0J	ACE2 / SARS-CoV-2 RBD	Infectious Disease	285
7C01	SARS-CoV-2 RBD / CB6 Antibody	Infectious Disease	324
7KMG	SARS-CoV-2 Spike / LY-CoV555	Infectious Disease	332

7 Heatmap of the effect of 6M0J complex mutations on binding free energy

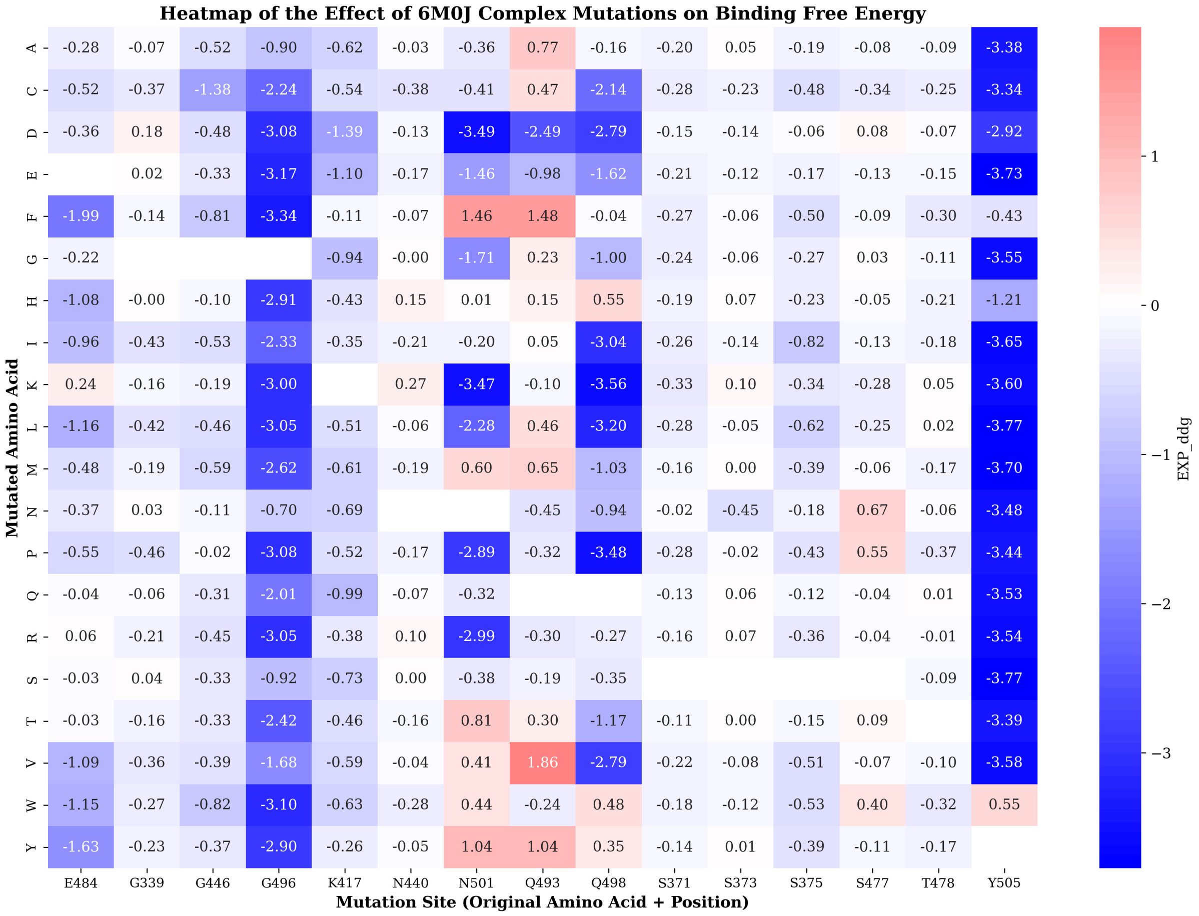

Figure 2: Heatmap of the saturation mutagenesis dataset for 15 sites in the 6M0J complex. Blue coloration indicates mutations that enhance binding affinity, while red coloration indicates mutations that weaken binding affinity.

8 Data distribution analysis for 6M0J, 7C01, 7KMG

Table 5: The percentage distribution of 6M0J, 7C01, and 7KMG across various datasets.

Dataset	Sample size	6M0J	7C01	7KMG
PPI_1753	1753	16.3%	18.5%	18.9%
PPI_single	1029	27.7%	1.5%	1.5%
PPI_multi	724	-	42.7%	43.8%
PPI_multi_2_5	410	-	37.6%	40.0%
PPI_multi_6	314	-	49.4%	48.7%
ABAG_921	921	-	35.2%	36.0%
ABAG_single	277	-	5.4%	5.4%
ABAG_multi	644	-	48.0%	49.2%
ABAG_multi_2_5	336	-	45.8%	48.8%
ABAG_multi_6	308	-	50.3%	49.7%

9 Analysis on single-point mutations located in the rigid regions of protein-protein interaction interfaces

We referenced a literature titled "Utility of B-Factors in Protein Science: Interpreting Rigidity, Flexibility, and Internal Motion and Engineering Thermostability", which explores the relationship between B-factors and rigid regions. Based on the conclusions of this literature, we conducted the following experiment.

First, for each protein complex in our dataset, we extracted B-factor values from the structural files (PDB format).

Then calculated the average B-factor value of all atoms (excluding hydrogen atoms) for each amino acid residue.

To enable comparison across different structures and chains, we normalized the B-factor for each amino acid in each chain to obtain a Z-score value, calculated using the following formula:

$$B\text{-factor}_{\text{norm}}(Z\text{-score}) = \frac{B_{\text{residue}} - B_{\text{chain}}}{\sigma_{B_{\text{chain}}}}$$

where B_{chain} is the mean and $\sigma_{B_{\text{chain}}}$ is the standard deviation of B-factors for all residues in that chain. If the Z-score value of the amino acid at the mutation site in a sample was less than 0, it was defined as a rigid region amino acid. Finally, we identified all single-point mutations in our dataset located in these rigid regions (Z-value < 0) and specifically re-evaluated the performance of all predictors on this subset of mutations (sample size N = 693).The performance metrics are presented in the table below.

Predictors	Pearson r	MAE	RMSE	Sample Size
Prompt	0.4213	1.1154	1.5881	693
Light	0.5453	1.0522	1.4723	693
DDMut-PPI	0.3555	1.1671	1.6814	693
GearBind	0.3549	1.1489	1.5892	693
DDAffinity	-0.0108	1.2365	1.7507	693
FoldX	-0.0112	1.8993	3.1301	693
SAAMBE-SEQ	-0.1985	1.4725	2.0226	661
